# An RGD-Containing Peptide Derived from Wild Silkworm Silk Fibroin Promotes Cell Adhesion and Spreading

**DOI:** 10.3390/polym10111193

**Published:** 2018-10-26

**Authors:** Zhao Kang, Yining Wang, Jingjing Xu, Guangzhou Song, Mengyao Ding, Huanrong Zhao, Jiannan Wang

**Affiliations:** 1 National Engineering Laboratory for Modern Silk, College of Textile and Clothing Engineering, Soochow University, No. 199 Ren-ai Road, Suzhou Industrial Park, Suzhou 215123, China; 20165215005@stu.suda.edu.cn (Z.K.); 20185215004@stu.suda.edu.cn (J.X.); 20185215032@stu.suda.edu.cn (G.S.); 20185215066@stu.suda.edu.cn (M.D.); zhaohuanrong1211@126.com (H.Z.); 2 Department of Biological Sciences, Xi’an Jiaotong-Liverpool University, No. 111 Ren-ai Road, Suzhou Industrial Park, Suzhou 215123, China; ywy960119@163.com

**Keywords:** wild silkworm, silk fibroin, RGD peptides, biosynthesis, cell adhesion, cell proliferation

## Abstract

Arginine-Glycine-Aspartate (RGD) tripeptide can promote cell adhesion when present in the amino acid of proteins such as fibronectin. In order to demonstrate the bioactivity of an RGD-containing silk protein, a gene encoding the RGD motif-containing peptide GSGAGGRGDGGYGSGSS (–RGD–) derived from nonmulberry silk was designed and cloned, then multimerised and inserted into a commercial pGEX expression vector for recombinant expression of (–RGD–)*_n_* peptides. Herein, we focus on two glutathione-S-transferase (GST)-tagged fusion proteins, GST–(–RGD–)_4_ and GST–(–RGD–)_8_, which were expressed in *Escherichia coli* BL21, purified by GST affinity chromatography, and analyzed with sodium dodecyl sulphate-polyacrylamide gel electrophoresis (SDS-PAGE) and mass spectrometry (MS). Target peptides (–RGD–)_4_ and (–RGD–)_8_ (6.03 and 11.5 kDa) were cleaved from the GST-tag by thrombin digestion, as verified with MS and SDS-PAGE. Isoelectric point analysis confirmed that target peptides were expressed and released in accordance with the original design. Target peptides self-assembled into a mainly α-helical structure, as determined by circular dichroism spectroscopy. Furthermore, (–RGD–)_4_ and (–RGD–)_8_ modified mulberry silk fibroin films were more effective for rapid cell adhesion, spreading and proliferative activity of L929 cells than some chemically synthesized RGD peptides modified and mulberry silk lacking the RGD motif.

## 1. Introduction

Silk fibroin is a natural protein synthesized and secreted by silkworms and spiders. *Bombyx mori* silk fibroin has been extensively used for biomaterials because it is readily obtainable on large quantities ad displays good biocompatibility and biodegradability, resulting in practicable cell adhesion and cell spreading, high proliferative and anti-thrombosis activities, and potent ability to induce tissue repair [1,2,3,4,5,6,7]. Wild silkworm species such as *Antheraea yamamai*, *Antheraea pernyi*, *Antheraea mylitta*, *Antheraea assama*, and *Philosamia ricini* secrete another type of silk fibroin with arginine-glycine-aspartate (RGD) motif repeats in the protein chain [8,9,10,11,12], which is absent in mulberry (*B. mori*) silk fibroin [13].

The RGD tripeptide is considered a recognition sequence for promoting cell adhesion, and was originally found in fibronectin, laminin, vitronectin, fibrin, and collagen, where it binds specifically to the cell surface [14]. RGD tripeptides in *A. yamamai* and *A. pernyi* silk fibroins have up to 14 and 12 repeats, respectively [8,9], and these silk fibroins display even higher cell affinity and hold greater promise for biomaterial applications than *B. mori* silk fibroin. Only a few studies have reported that regenerated RGD tripeptide-containing silk fibroin materials from wild silkworm species are potentially useful biomaterials, with satisfactory cytocompatibility and the ability to promote tissue remodeling [15,16,17]. However, the behaviors of wild silkworm species renders them unsuitable for domestication, resulting low production and minimal scope for biomaterial applications.

To provide a theoretical foundation for increasing the applicability of nonmulberry silk fibroins to particular cell lines and tissue engineering, RGD-containing multimers (–RGD–)_4_ and (–RGD–)_8_ based on the monomer GSGAGGRGDGGYGSGSS derived from *A. pernyi* or *A. yamamai* silk fibroins were recombinantly produced in *Escherichia coli* BL21. Their effects on cell behavior were preliminarily evaluated following grafting onto mulberry (*B. mori*) silk fibroin films using L929 cells.

## 2. Materials and Methods

### 2.1. Protein Expression 

Expression vectors pGEX–AY(*n*), carrying the AY1 gene motif encoding RGD-containing peptide GSGAGGRGDGGYGSGSS (–RGD–) derived from nonmulberry silk, and its multimers AY(*n*), were constructed as previously reported [18,19]. The two vectors pGEX–AY(4) and pGEX–AY(8) were separately transformed into *E. coli* BL21 (DE3) cells to express glutathione-S-transferase (GST)-tagged fusion proteins GST–(–RGD–)_4_ and GST–(–RGD–)_8_, respectively. Different initial cell densities (OD_600_ = 0–2.1 AU, at 0.3 AU intervals) and different isopropyl–β–d–thiogalactoside (IPTG) concentrations (0–1.0 mM, at 0.2 mM intervals) were tested to optimize the expression levels of the two fusion proteins. At different time points between 1 and 8 h following IPTG induction, cells were harvested by centrifugation at 4 °C and stored at −80 °C.

### 2.2. Protein Purification

Fusion proteins were purified using a GST affinity purification system (Novagen, Billerica, MA, USA) as previously described [20]. Briefly, the cell pellet was suspended in GST-bind/wash buffer and sonicated on ice. The lysate was centrifuged at 4 °C and the supernatant was loaded onto a GST affinity column and washed with GST-wash buffer. Finally, the fusion protein was eluted with GST-elution buffer, then loaded onto a Sephadex G-15 zeolite column (Solarbio, Beijing, China) to remove glutathione and salt.

### 2.3. Determination of Expression Yield 

The yield of purified fusion protein was determined using a Smartspec Plus UV/visible spectrophotometer (Bio-Rad, Hercules, CA, USA) by measuring the absorbance at 260 and 280 nm. Protein concentration was calculated using the formula C (mg/mL) = (1.45 × *A_280_*) − (0.74 × *A_260_*), then converted to the amount per L of bacterial cell culture [21].

### 2.4. Cleavage of Fusion Proteins

The (–RGD–)_4_ and GST–(–RGD–)_8_ peptides from fusion proteins were obtained, as described previously [20]. Briefly, purified fusion protein was digested with thrombin (Novagen) at 20 °C for 16 h, and the reaction mixture was loaded onto a GST affinity column to remove the GST-tag. Finally, samples were freeze-dried and stored at 4 °C.

### 2.5. Molecular Weight Determination

Molecular weight was determined with sulphate-polyacrylamide gel electrophoresis (SDS-PAGE) and mass spectrometry (MS) as described previously [20]. Briefly, cell lysate or purified fusion protein was mixed with loading buffer and boiled for 3–5 min, then loaded onto a 10% (*w*/*v*) polyacrylamide gel (Sigma, St. Louis, MO, USA) and stained using Coomassie Brilliant Blue. Molecular weight was qualitatively analysed by referring to protein molecular weight standards. Quantitative analysis of molecular weight was performed using a 4800 MALDI-TOF/TOF mass spectrometer (AB SCIEX, Foster City, CA, USA).

### 2.6. Charge Assay 

The ζ-potential of fusion proteins or liberated peptides was measured using a ZS90 Zetasizer Nano (Malvern Instruments, Malvern, UK) in 5 mM sodium phosphate buffer with 5 mM NaCl at 25 °C. The pH of the buffer was adjusted to 5.0, 6.0, 7.0, 8.0, 9.0, and 10.0 using NaOH or HCl.

### 2.7. Circular Dichroism (CD) Assay

The ellipticity of 0.1 mg/mL purified (–RGD–)_4_ and (–RGD–)_8_ peptide solutions was measured using a J-815 CD spectrometer (Jasco, Tokyo, Japan) with a 1.0 mm path-length cell at 25 °C, an accumulation time of 4 s, and a scanning rate of 100 nm/min. A blank solution was measured under the same conditions and subtracted from sample spectra.

### 2.8. Preparation of (–RGD–)_n_-Modified Mulberry Silk Fibroin Films

*B. mori* silk fibroin solution was prepared as described previously [22]. Silk fibroin films were obtained by casting 300 μL of 4% (*w*/*v*) silk fibroin aqueous solution onto each well of a 24-well tissue culture plate, drying at 60 °C, then treating with 80% ethanol for 30 min. Silk fibroin films were dipped in 0.05 M2-morpholinoethane sulfonic acid (pH 5.5) and acidified for 1 h on ice, and 1 mL of 0.5 mg/mL 1-ethyl-3-(3-dimethyl aminopropyl)-carbodiimide hydrochloride was then added to each well and incubated for 1 h. Peptides (–RGD–)_4_, (–RGD–)_8_, GRGDS, GGRGDGGYGS (RGD-10), and GGRGEGGYGS (RGE-10) were added into each well, followed by 0.5 mL of 0.7 mg/mL *N*-hydroxysuccinimide. After reaction overnight at 4 °C, reaction mixtures were removed and films were washed three times with phosphate-buffered saline (PBS; pH 7.4), air dried, and sterilized by Co^60^ for cell culturing. Peptides GRGDS, RGD-10, and RGE-10 were purchased from GL Biochemistry Limited (Shanghai, China).

### 2.9. Cell Adhesion Assay

L929 fibroblasts were cultured in Dulbecco’s modified Eagles medium (Gibco, Carlsbad, CA, USA) containing 10% (*v*/*v*) fetal bovine serum (Gibco, Carlsbad, CA, USA) and 1% (*v*/*v*) antibiotics (100 U/mL penicillin and 100 μg/mL streptomycin) at 37 °C in a 5% CO_2_ incubator. During the logarithmic growth phase, cells were trypsinized using 0.25% trypsin (Sigma, St. Louis, MO, USA) and resuspended at a density of 1.0 × 10^5^ cells/mL. A 1 mL sample of the L929 cell suspension (1.0 × 10^5^ cells, N_1_) was added to each well pre-coated with (–RGD–)_4_– or (–RGD–)_8_-modified mulberry silk fibroin films and incubated at 37 °C in 5% CO_2_. After seeding for 1, 2, or 3 h, loosely adhered or unattached cells were removed and films were carefully washed twice with PBS (pH 7.4). The cell number (N_2_) in residual liquid was counted using a haemocytometer and an inverted microscope (TH4-200, Olympus, Tokyo, Japan), and converted into cell adhesion rate. The cell adhesion ratio was calculated using the following equation:

(1) $$\mathrm{Adhesion}\;\mathrm{ration}\;(\%)\;=\;\frac{N_{1}-N_{2}}{N_{1}}\;\times \;100\%$$

### 2.10. Cell Viability Evaluation

Cells (2.5 × 10^4^ cells/well) were added to 24-well tissue culture plates coated with peptide-modified films and incubated at 37 °C in 5% CO_2_. After culturing for 1 or 3 days, cell morphology was observed using the TH4-200 inverted microscope. On day 3, the cell count was determined using the haemocytometer. The cell proliferation ratio was calculated, as described in Section 2.9. Half of the medium was replaced every other day.

## 3. Results 

### 3.1. SDS-Page Analysis of Total Protein from E. coli BL21 Cells

**Figure 1:**
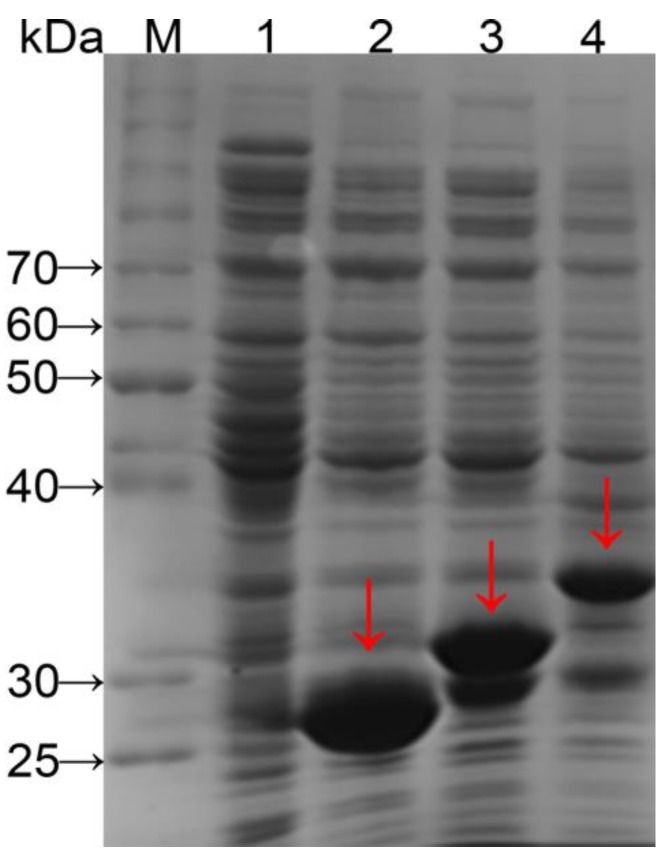
SDS-PAGE of fusion proteins tentatively expressed in *E. coli* BL21. lane M, protein molecular weight standards; lane 1, not containing the expression vector; lane 2, containing the expression vector pGEX-AgeI [18]; lane 3, containing the expression vector pGEX–AY(4); and lane 4, containing the expression vector pGEX–AY(8).

Genes were expressed under the control of the Ptac promoter with a translation-enhancing sequence (g10) and a ribosome-binding site for regulation of translation level. A GST-tag was used to purify expression products and a protease (thrombin) recognition site (Leu-Val-Pro-Arg-Gly-Ser) was inserted to enable target peptide release from the GST-tag by cleaving the amide linkage between Arg and Gly [20]. Fusion proteins GST–(–RGD–)_4_ and GST–(–RGD–)_8_ from crude cell extracts were analyzed with SDS-PAGE (Figure 1) and bands of a size close to the expected molecular weight (32.1 and 37.7 kDa, respectively) were observed (lanes 3 and 4). The molecular weights were further confirmed by MS.

**Figure 2:**
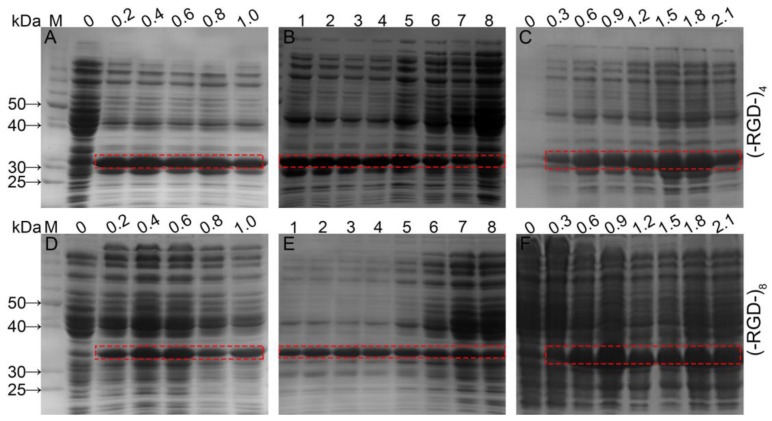
Fusion proteins expression under different conditions. (**A**,**D**), different IPTG concentrations (mM); (**B**,**E**), different induction times (h); (**C**,**F**), different initial cell densities (AU); and lane M, proteinmolecular weight standards.

Expression levels of fusion proteins were optimized by regulating IPTG concentration, induction time, and initial cell density (Figure 2). When the initial cell density reached OD_600_ = 0.6 AU, protein expression was induced by 0.1–1.0 mM IPTG and culturing continued for up to 6 h at 37 °C with shaking. Bands corresponding to fusion proteins were clearly visible following induction with 0.2 mM IPTG, and the optimal IPTG concentration was 0.4 mM for GST–(–RGD–)_4_ and 0.4–0.6 mM for GST–(–RGD–)_8_. Expression of target proteins was minimal without IPTG induction, but some *E. coli* proteins displayed high expression. Using 0.4 mM IPTG and an OD_600_ = 0.6 AU, expression of both fusion protein variants was enhanced by extending the induction time. Following a 1 h induction, the GST–(–RGD–)_4_ expression level increased markedly, with no subsequent change until 5 h post-induction. After that, expression of *E. coli* proteins increased while the GST–(–RGD–)_4_ expression level gradually decreased. GST–(–RGD–)_8_ expression levels were already appreciable following a 1 h induction and peaked after 3–4 h, but expression of *E. coli* proteins increased markedly after a 6 h induction. The optimal pre-induction density was OD_600_ = 1.5 AU for GST–(–RGD–)_4_ expression, and OD_600_ = 0.9 AU for GST–(–RGD–)_8_ expression.

### 3.2. SDS-PAGE and MS Analysis of Purified Fusion Proteins

**Figure 3:**
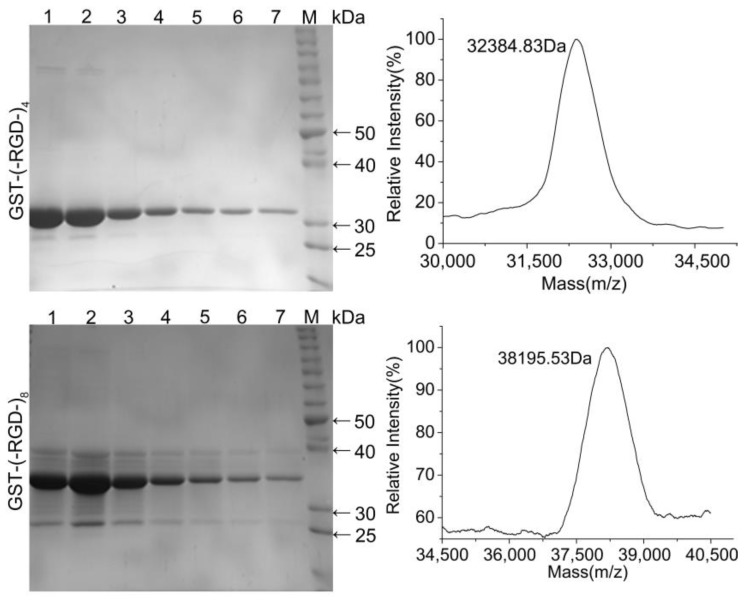
SDS-PAGE (**Left**) and MS (**Right**) graphs of purified fusion proteins. Lane 1–7, collected in successive tubes (1 mL/tube); and lane M, protein molecular weight standards.

Protein purification was expedited by the GST-tag encoded in the pGEX-KG vector. SDS-PAGE analysis confirmed the high purity of the fusion proteins following affinity chromatography (Figure 3). Both fusion proteins were highly stable with no obvious degradation, and the molecular weights were similar to the predicted values of 32.1 and 37.7 kDa. Accurate molecular weights determined for GST–(–RGD–)_4_ and GST–(–RGD–)_8_ by MS were 32.86 and 38.42 kDa, close to the predicted values. These results confirmed the successful expression and purification of each variant.

Using the optimized expression conditions, the expression yield for GST–(–RGD–)_4_ was 55 ± 0.08 mg per l of BL21 cells after induction for 2 h with 0.4 mM IPTG at an initial cell density of 1.5 AU. For GST–(–RGD–)_8_ the yield was 52.5 ± 0.2 mg per l of BL21 cells after induction for 3 h with 0.4 mM IPTG at an initial cell density of 0.9 AU.

### 3.3. SDS-PAGE and MS Analysis of Released Peptides

**Figure 4:**
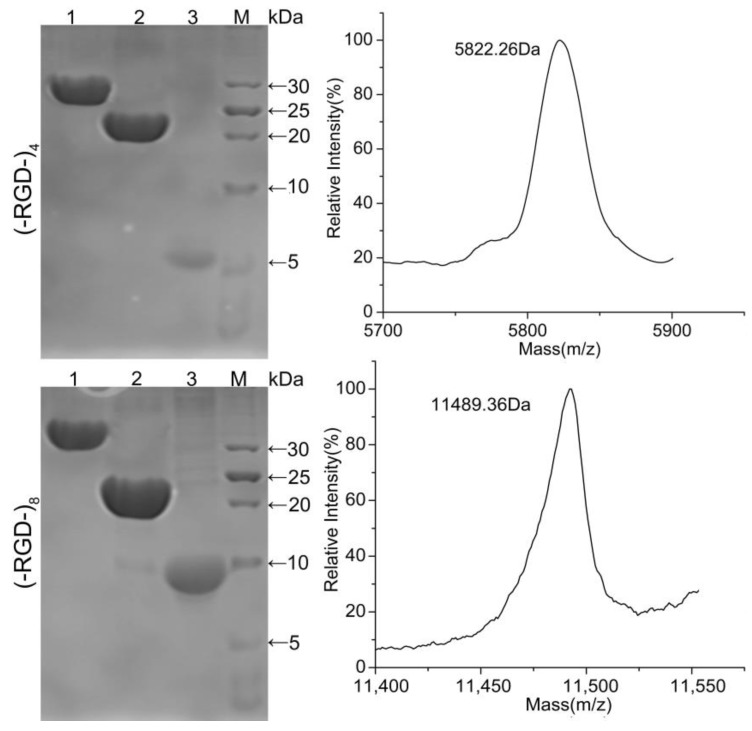
SDS-PAGE (**Left**) and MS (**Right**) of released peptides. Lane 1, fusion proteins GST–(–RGD–)_4_ (Up) and GST–(–RGD–)_8_ (Down); lane 2, peptides (–RGD–)_4_ (Up) and (–RGD–)_8_ (Down); lane 3, GST tag; and lane M, protein molecular weight standards.

Fusion proteins GST–(–RGD–)_4_ and GST–(–RGD–)_8_ were incubated with thrombin and purified using GST affinity chromatography to obtain target peptides (–RGD–)_4_ and (–RGD–)_8_. As shown in Figure 4, GST–(–RGD–)_4_ and GST–(–RGD–)_8_ (lane 1) were efficiently cleaved into two fragments, GST (lane 2) and target peptide (lane 3). The bands appearing in lane 3 qualitatively indicated the successful expression of (–RGD–)_4_ and (–RGD–)_8_ according to protein molecular weight standards, and molecular weights were confirmed using MS, which were 5.726 and 11.498 kDa for (–RGD–)_4_ and (–RGD–)_8_, respectively, consistent with the predicted values 6.03 and 11.5 kDa. The results of SDS-PAGE and MS confirmed that the target peptides were correctly expressed, highly purified, and stable.

### 3.4. CD Analysis of Released Peptides

**Figure 5:**
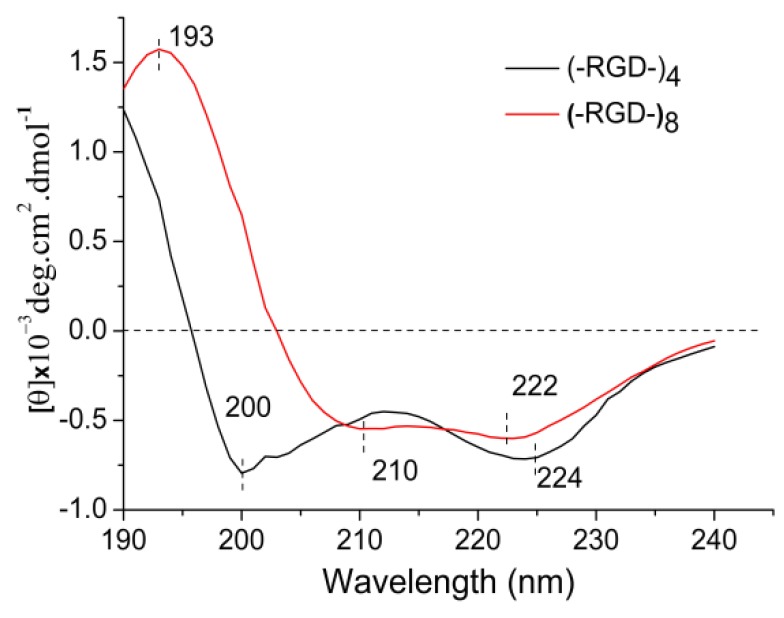
CD spectra of released peptides.

The functions of bioactive macromolecules depend on their structures. The molecular conformation of a protein presents characteristic absorption peaks in the far-UV area (170–250 nm) of CD spectra. A strong negative cotton effect peak at 195–202 nm is characteristic of random coil structure. A strong positive cotton effect peak at 185–200 nm and a negative wide peak around 217 nm are characteristic of β-sheet structure. The characteristic peaks of α-helical structure include a positive cotton effect peak around 192 nm, a negative cotton effect peak at 207–208 nm, and/or a negative peak around 222 nm. Negative cotton effect peaks around 192.5 nm and 227 nm, and a positive cotton effect peak at 200–205 nm indicate β-turn structure. Figure 5 shows the spectrum of (–RGD–)_4_ with strong negative cotton effect peaks around 200 nm assigned to random coil, and a 224 nm peak assigned to α-helical or transitional β-turn structures. The spectrum of (–RGD–)_8_ included characteristic peaks of α-helical structure at 210 nm (negative), 222 nm (negative) and 193 nm (positive). Thus, the CD spectra suggest that the molecular conformation changed as the molecular chain was lengthened. The peptide GSGAGGRGDGGYGSGSS (–RGD–) contains hydrophilic side groups –OH, –NH_2_ and –COOH, hence it is easier for peptides with a greater number of –RGD– repeats to form intramolecular hydrogen bonds, leading to a change in molecular conformation into a more stable α-helical structure in (–RGD–)_8_. Many bioactive macromolecules include a high proportion of α-helical structure, and this is often correlated with physicochemical properties [23,24].

### 3.5. Charge Analysis of Released Peptides

**Figure 6:**
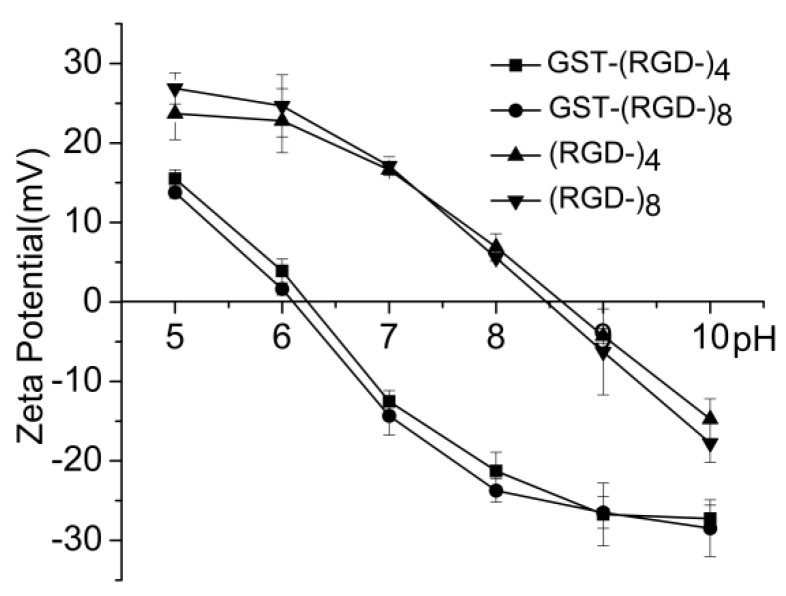
Charge characteristic of expression products.

Amphoterism is an important characteristic of a protein. The fusion proteins in this study exhibited a negative ζ-potential in neutral aqueous solution, and the measured isoelectric point (pI) for both GST–(–RGD–)_4_ and GST–(–RGD–)_8_ was between 6.2 and 6.5 (Figure 6), consistent with the predicted value of 6.61. After digestion, peptides released from fusion proteins exhibited a positive ζ-potential in neutral aqueous solution, and the measured pI values for (–RGD–)_4_ and (–RGD–)_8_ were 8.7 and 8.5, respectively, which are very close to the predicted values of 8.72 and 8.55.

### 3.6. Cell Adhesion and Proliferation

**Figure 7:**
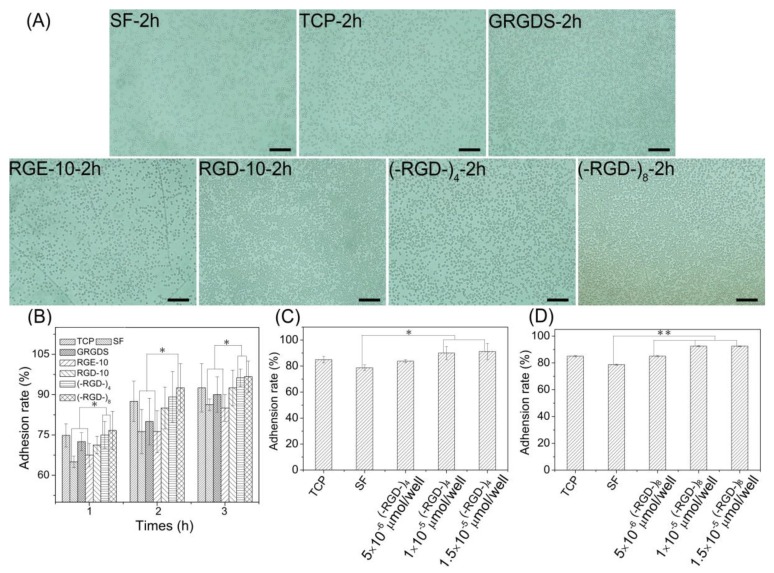
Cell adhesion property on RGD modified mulberry silk fibroin films. (**A**) Cell adhesion photographs on the different materials; (**B**) cell adhesion rate on the different materials (1 × 10^−5^ moL/cell of peptides); (**C**) cell adhesion rate on mulberry silk fibroin films modified with (–RGD–)_4_ in different dose; (**D**) cell adhesion rate on mulberry silk fibroin films modified with (–RGD–)_8_ in different doses; C and D: after 2 h of cells seeding; and TCP, tissue cell plate; SF, silk fibroin. * *p* < 0.05, ** *p* < 0.01, *n* = 3.

Cell adhesion and cell proliferation activities of (–RGD–)_4_– and (–RGD–)_8_-modified mulberry silk fibroin films were evaluated by seeding L929 cells. At 1 h after seeding, more than 60% of L929 cells adhered stably to all materials (Figure 7A). Pure mulberry silk fibroin film was the most unfavorable for cell adhesion; the cell adhesion rate on RGE-10-modified mulberry silk fibroin film was close to that on unmodified mulberry silk fibroin film. After modification with GRGDS and RGD-10, the cell adhesion rate increased relative to that on unmodified mulberry silk fibroin film, and films modified with (–RGD–)_4_ or (–RGD–)_8_ were more favourable for cell adhesion (Figure 7B), and the increase in the cell adhesion rate was concentration-dependent. Maximal cell adhesion rates on silk fibroin films were detected at a 0.01 μmoL/cm^2^ dose of (–RGD–)_4_ and a 0.005 μmoL/cm^2^ dose of (–RGD–)_8_ (Figure 7C,D), and differences compared to unmodified mulberry silk fibroin film were significant. 

**Figure 8:**
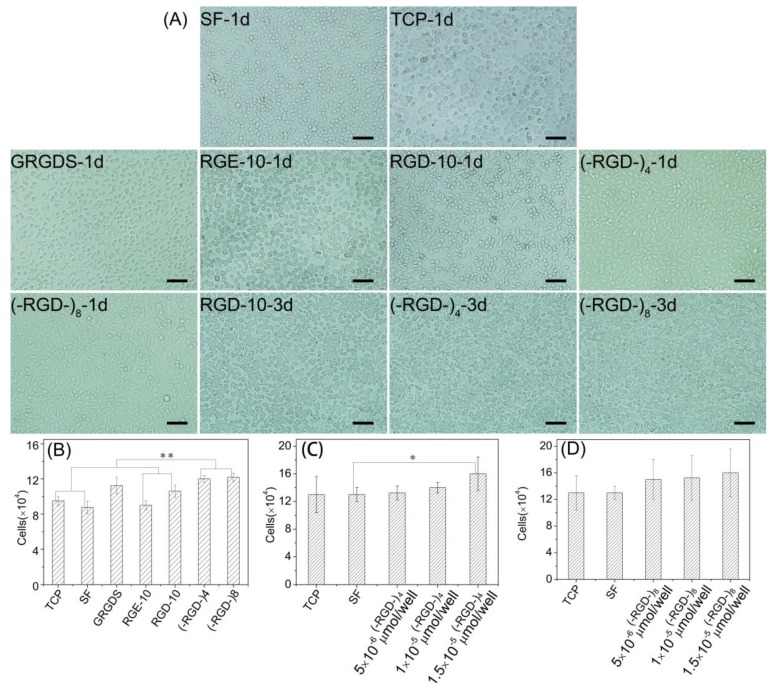
Cell morphology and cell proliferation activity on RGD modified mulberry silk fibroin films. (**A**) Cell morphology; (**B**) cell proliferation rate on the different materials (1 × 10^−5^ mol/cell of peptides); and (**C**) cell proliferation rate on mulberry silk fibroin films modified with (–RGD–)_4_ in different doses. (**D**) Cell proliferation rate on mulberry silk fibroin films modified with (–RGD–)_8_ in different doses; (**B**–**D**): after 3 days of cells seeding; TCP, tissue cell plate; SF, silk fibroin. * *p* < 0.05, ** *p* < 0.01, *n* = 3.

Furthermore, L929 cells underwent better spreading into spindle, triangular, or polygonal shapes on (–RGD–)_4_–, (–RGD–)_8_–, and RGD-10-modified mulberry silk fibroin films compared with other samples on day 1, and spindle cells were particularly prevalent in (–RGD–)_4_ and (–RGD–)_8_ samples. Cells showed satisfactory proliferation activity, fully covered samples, and many cells were beginning to emerge at 3 days after seeding (Figure 8A). The cell proliferation activity on mulberry silk fibroin films modified with (–RGD–)_4_ (*p* < 0.01) or (–RGD–)_8_ (*p* < 0.01) was higher than that on unmodified film, cell culture plate controls, and the RGE-10 sample, and was also higher than on any other modified materials (Figure 8B). However, there was no significant difference in cell proliferation rate on (–RGD–)_4_– or (–RGD–)_8_-modified mulberry silk fibroin films compared with film modified with the chemosynthetic peptide GRGDS. After modification by (–RGD–)_4_ and (–RGD–)_8_ at a same dose of 0.015 μmoL/cm^2^, the cell proliferation rate on mulberry silk fibroin films was increased significantly (Figure 8C,D), but a further increase in the dose did not increase cell proliferation any further.

## 4. Discussion

RGD repeat motifs are present in fibronectin, a well-characterized extracellular glycoprotein that interacts strongly with other extracellular matrix molecules to promote cell adhesion and spreading, and this tripeptide motif is also present in some other extracellular matrix proteins. RGD peptides have been used to modify synthetic polymers to manipulate cell behavior, promote cell adhesion, proliferation and spreading, and induce stem cell differentiation [25,26,27,28,29]. When encapsulated within three-dimensional hydrogel systems, RGD peptide presentation can guide cell motility of encapsulated cells [30,31,32]. In recent oncotherapy research, RGD-decorated nanoparticles were shown to enhance cell targeting and uptake, leading to more effective anti-tumor effects and demonstrating high potential for targeted chemotherapy of cancer cells [33,34,35,36].

Not all RGD tripeptide-containing peptides or proteins can promote cell adhesion. For example, Arg-Gly-Asp-Thr-Gly-Ala-Thr-Gly-Arg (derived from type I collagen) promotes cell adhesion, while Glu-Gly-Ile-Arg-Gly-Asp-Lys-Gly-Glu-Pro and Gly-Ser-Arg-Gly-Asp-Hyp-Gly-Thr-Hyp (derived from collagens of different genetic types) do not [37]. RGD tripeptide-containing nonmulberry silk fibroins are potential biomaterials that may possess better cell-binding ability than mulberry silk fibroins without this motif, although the RGD sequence has not previously been verified to promote cell adhesion. Herein, we developed the GSGAGGRGDGGYGSGSS peptide (–RGD–, completely derived from nonmulberry silk fibroin) and multimers thereof (–RGD–)*_n_* at less cost, which were reported in previous studies [19] by efficient recombinant expression in *E. coli*. Our aim was to determine whether nonmulberry silk fibroins could affect cell responses, and therefore have potential for applications in stem cell differentiation, tissue repair, or oncotherapy.

**Figure 9:**
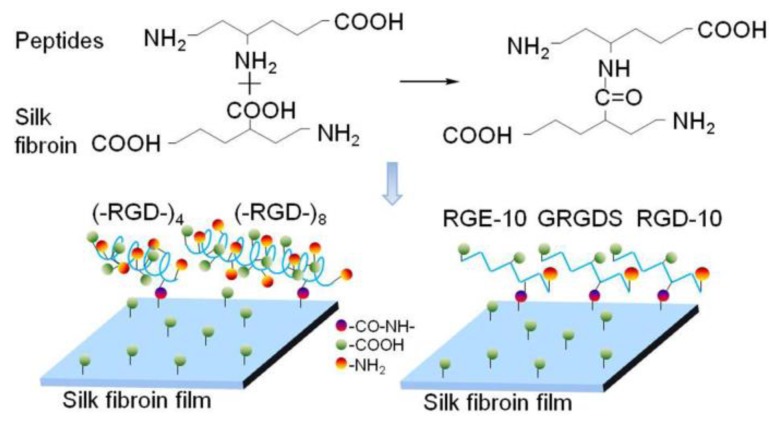
Reaction principle of RGD peptides grafting onto mulberry silk fibroin.

The structure and properties of all proteins is dependent on the nature and distribution of amino acid side chains. As shown in Figure 7, mulberry silk fibroin was no more favorable for attachment of L929 cells than the bare cell culture plate. After grafting GRGDS and RGD-10 peptides, the adherence rate of L929 cells was increased, but the effect was not pronounced. By contrast, the adhesion ability was improved significantly when (–RGD–)_4_ or (–RGD–)_8_ were grafted onto the mulberry silk fibroin surfaces, to levels higher than on bare cell culture plates. Meanwhile, RGE-10 displayed no cell adhesion-promoting activity, similar to unmodified mulberry silk fibroin when Aspartate residues were substituted by glutamate. In our experiments, all peptides were grafted onto mulberry silk fibroin through amide bonds formed by the –COOH groups of silk fibroin and the –NH_2_ groups of peptide Arg residues (Figure 9). The cell adhesion-promoting activity was increased after coupling GRGDS and RGD-10 peptides to mulberry silk fibroin by only the –NH_2_ groups, compared with ungrafted mulberry silk fibroin. However, cell adhesion rates were lower than cell culture plate controls. Interestingly, because there are four or eight repeats of the RGD tripeptide in the peptide chain of (–RGD–)_4_ and (–RGD–)_8_), some –NH_2_ groups remained unoccupied when grafted to mulberry silk fibroin, resulting in significantly improved cell adhesion activity compared with ungrafted mulberry silk fibroin, above those of the bare cell culture plate. These results are consistent with a previous report [37], and suggest that only the highly conserved RGD tripeptide sequence can promote cell adhesion activity, since cell binding activity was lost or decreased when arginine or aspartate residues were replaced.

RGD-containing peptides have been widely used to modify polymers by covalent binding via reaction with –COOH groups of Asp and/or –NH_2_ groups of Arg. However, our results showed that the RGD tripeptide and free side –COOH and –NH_2_ groups had a profound impact on cell adhesion. In order to react RGD-containing fibroin proteins from wild silkworms more efficiently, we should explore new pathways to investigate their bioactivity and cell responses.

RGD-containing peptides have distinct effects on cells from different species. Metastatic cells attach preferentially to type IV collagen, and laminin can increase both the rate and number of metastatic cells attaching to type IV collagen, while fibronectin has no such effect [38]. By contrast, fibronectin can promote the cell adhesion activity of fibroblasts, while laminin has no such effect [39]. As shown in Figure 7 and Figure 8, adhesion rates on mulberry silk fibroin films grafted to the RGD-10 decapeptide were somewhat higher than those grafted to GRGDS, but this did not enhance the cell proliferation rate; there was significant differences in cell proliferation rate of (–RGD–)_4_– or (–RGD–)_8_-modified mulberry silk fibroin films compared to cell culture plate, but was not in the cell adhesion rate. This suggests that cellular response mechanisms are complex and related to specific structural domains, and sequences flanking the Arg-Gly-Asp (-Ser) sequence also affect activity [37].

## 5. Conclusions

Herein, the RGD motif-containing peptide GSGAGGRGDGGYGSGSS (–RGD–) derived from *A. pernyi* and *A. yamamai* was recombinantly expressed and purified, and confirmed by MS, amino acid composition analysis, and SDS-PAGE. The resulting (–RGD–)_4_ and (–RGD–)_8_ target peptides promoted cell adhesion to materials and cell spreading, but had no greater effect on cell proliferation than chemically synthesized RGD-containing peptides. We preliminarily evaluated the cytocompatibility of L929 cells related to the recombinant RGD-containing peptides, and in future work we intend to investigate cell behavioral responses to RGD-containing peptides derived from nonmulberry silk fibroin using a variety of systems for probing adhesion, proliferation, and migration of various cell types, differentiation of stem cells, and target uptake of tumor cells.

## Abbreviations

**Amino Acids**	
G	Gly
S	Ser
A	Ala
R	Arg
D	Asp
Y	Tyr
